# A nanobody-derived mimotope against VEGF inhibits cancer angiogenesis

**DOI:** 10.1080/14756366.2020.1758690

**Published:** 2020-05-22

**Authors:** Elmira Karami, Jean-Marc Sabatier, Mahdi Behdani, Shiva Irani, Fatemeh Kazemi-Lomedasht

**Affiliations:** a Venom and Biotherapeutics Molecules Laboratory, Biotechnology Department, Biotechnology Research Center, Pasteur Institute of Iran, Tehran, Iran; b Department of Biology, Science and Research Branch, Islamic Azad University, Tehran, Iran; c Aix-Marseille Université, Institut de Neurophysiopathologie (INP) UMR 7051, 27 boulevard Jean Moulin, Faculté de Médecine, 13385 - Marseille Cédex 5, France

**Keywords:** Angiogenesis, VEGF, nanobody, mimotope, peptide

## Abstract

Vascular Endothelial Growth Factor (VEGF) promotes angiogenesis in tumours of various cancers. Monoclonal antibodies and nanobodies are one of the potent agents in the treatment of cancer. Due to their high costs, researchers are considering to design and produce peptides as a substitute approach in recent years. The aim of the current study was designing a mimotope against VEGF and evaluate its effects on cell proliferation and tube formation in the HUVEC cell line. For this, a peptide was designed against VEGF and chemically produced. The effects of synthetic peptide and nanobody on the inhibition of proliferation of HUVEC cells were examined using MTT and tube formation assays. The data indicate that the peptide was able to significantly inhibit both HUVEC cell proliferation and tube formation through inhibition of VEGF, highlighting the potential of peptides as a ‘novel’ class of candidate drugs to inhibit angiogenesis.

## 1. Introduction

Angiogenesis is a natural process to form new blood vessels. Activities like reproduction, embryonic growth and wound healing actually depend on the formation of new blood vessels. In addition, aberrant angiogenesis is involved in malignancies like cancer. Vascular endothelial growth factor (VEGF) is the most important and specific angiogenesis factor of a tumour, and excessive angiogenesis leads to the tumour development^1^. The VEGF family includes VEGF-A, VEGF-B, VEGF-C, VEGF-D, and PIGF^2^. Folkman suggested that utilising anti-angiogenesis compounds would be a promising approach in cancer treatment^3^. In 1975, George Kohler and Cesar Milstein – by introducing the relevant hybridoma technique – led to a huge change in the production of monoclonal antibodies. At least three decades ago started the studies about utilising monoclonal antibodies as therapeutic compounds for treatment of cancer^4^. Beside monoclonal antibodies, nanobodies appear to be a new generation of antibody-like compounds with properties and potentials similar to those of monoclonal antibodies and small drugs^5^. Hamers-Casterman was first to discover the nanobodies in 1993^6^. In 2008, Roider found that ranibizumab was effective in cancer treatment; however, because of its side effects, Kolkman started to produce nanobodies^6^^,^^7^. Kazemi et al. also developed nanobodies against VEGF that were able to inhibit VEGF^5^^,^^8^^,^^9^. The nanobodies have a number of advantages, which include the potentially high affinities towards their molecular targets and the intrinsic low immunogenicities^10–15^. In addition, they often behave as highly soluble and stable compounds which, according to their small sizes, can bind to regions/domains of antigens which are inaccessible to other common types of antibodies^16^. Generally, nanobodies have accurate folding and are highly expressed in bacterial and yeast hosts^15^^,^^17^^,^^18^. The VHH or nanobody is a single domain antibody derived from the heavy chain’s variable region of an antibody^19^. Previous studies have demonstrated that VHH activity relies on its CDR3 region. Also, long CDR3 sequences enable it to better bind to the active site of enzymes^20^^,^^21^. Some peptide-based drugs have caught particular attention because of their abilities to ‘compensate’ therapeutic failings, as well as their small sizes and relative accessibility^22^. The mimotopes or peptidomimetics are small peptides that are recognised by the human immune system, and which possess some ‘key’ structural features resembling those of the antibody binding sites^23^^,^^24^. The mimotopes are peptides ‘mimicking’ proteins. Mimotopes represent new approaches in the treatment of human disorders such as cancer^25^. In 2017, Pourhashem et al. designed and produced a peptidomimetic (named HER3) from its nanobody^24^. Mimotopes have many advantages such as an enhanced stability and a possible large-scale production. In addition, mimotopes can be stored as freeze-dried powders for long periods. They can bind to immune carriers and enhance immunity^25^^,^^26^.

In the present study, we aimed to design a peptidomimetic targeting VEGF from its nanobody that can inhibit VEGF, and consequently angiogenesis. The CDR3 region of nanobody shows an affinity to VEGF similar to that of the entire nanobody, whereas the peptide and nanobody showed similar effects in functional assays.

## 2. Materials and methods

### 2.1. Bioinformatics studies

Anti VEGF nanobody was from previous study named Nb42^27^. Anti-angiogenesis activity of Nb42 (here named VEGF nanobody) were studied *in vitro* and *in vivo*^28^. A primary structure derived from the VEGF nanobody was submitted to the IMGT database in order to detect its CDR fragments^29^. After detecting the CDR fragments, some more ‘complex’ structures of VEGF nanobodies were designed using the I-TASSER Swiss homology modelling database^30^. In order to optimise the 3 D structures of each CDR, SPDU swiss viewer database was used^31^^,^^32^. Furthermore, docking simulation experiments were performed for the predicted structures using Hex software, and the final selected structure was chemically synthesised^33^. To investigate the interactions between nanobody’s CDRs and VEGF, the protein–protein interaction method was used. In docking simulation experiments, VEGF-A was the receptor whereas the CDR structures were considered to be the ligands. The results of docking simulation experiments were finally analysed by the Hex software.

### 2.2. Peptide design and chemical synthesis

According to the data obtained by docking simulation experiments, the theoretically more ‘appropriate’ CDR3 region of nanobody was selected and chemically produced by solid-phase peptide synthesis. The designed 25-mer peptide (carboxyl-amidated at C-terminus; -CONH2) possesses the following amino acid sequence (one-letter code, IUPAC convention):

YY(Abu)AARAWSPYSSTVDAGDFRYWGQ-NH2, where “(Abu)” stands for alpha-amino-butyrate (an isosteric analogue of half-cystine residue with a side-chain methyl (-CH3) replacing the thiol (-SH) group).

### 2.3. Recombinant VEGF nanobody expression and purification

A colony of *E. coli* WK6 bacteria carrying the VEGF nanobody recombinant gene was cultured in LB media. Bacteria were treated with different concentrations of IPTG (Isopropylβ-D-1-ThioGalactopyranoside) in their logarithmic phase (OD_600nm_ 0.4– 0.6) and were incubated at a temperature of 30 °C at 180 rpm. After a 16 h-incubation period, the pellet of bacteria was suspended in 12 ml of TES (0.2 M Tris, 0.5 mM EDTA, 0.5 M Sucrose) buffer and incubated for 1 h at a temperature of 4 °C. Then, 18 ml of TES/4 were added and incubation was continued (temperature of 4 °C for 1 h). Then, a centrifugation at 10,000 x*g* was performed for 30 min. The supernatant was finally loaded onto a nickel affinity column) Ni-NTA) (QIAGEN, Germany) pre-equilibrated with the washing buffer (Tris 50 mM, Imidazole 10 mM, NaCl 500 mM). The recombinant protein fraction was eluted from the column using PBS buffer plus Imidazole 250 mM, and its concentration was assessed by using the nanodrop spectrophotometer (Epoch). The high degree of purity of the recombinant protein was confirmed by SDS-PAGE and western blotting (15% polyacrylamide gel). For western blotting, protein bands were transferred to the nitrocellulose surface using 4% skim milk (Merck) followed by an overnight incubation at a temperature of 4 °C. Then, the primary antibody (Anti-His antibody) (1:2000) was added and incubated overnight. Subsequently, the secondary antibody (anti-human HRP-conjugated antibody (1:1000) was added and incubated for 6 h. Finally, colouring dye 1 (methanol + 4 chlore 1- nephtol) and colouring dye 2 (H_2_O_2_ + PBS buffer) were added to the nitrocellulose surface, followed by an incubation in darkness for 15 min.

### 2.4. Affinity analysis

Affinity of designed peptide as well as nanobody to VEGF was calculated according to Beatty et al. method using below equation^34^:

$$[\mathrm{Ag}]/[{\mathrm{Ag}}^{\prime }]=N$$

$$K_{\mathrm{aff}}=N-1/2(N[\mathrm{Nb}]-[{\mathrm{Nb}}^{\prime }])$$

Briefly, two different concentrations of VEGF (1 and 10 μg/ml) was coated on 96-well plate at 4 °C overnight. Next day, the wells were blocked with skim milk 4% and incubated at RT for 2 h. After removing the blocking buffer, serial dilutions of peptide, nanobody, BSA (control), and Bevacizumab (positive control) (0–100 nM) were added to the wells and incubated at RT for 1 h. Binding of peptide, nanobody, and Bevacizumab to VEGF was detected by rabbit anti-peptide (developed in our lab) followed by anti rabbit HRP conjugated, anti-His HRP conjugated, and anti-human Fc HRP conjugated, respectively. Peroxides activity was monitored by TMB.

### 2.5. Huvec cell culture

The HUVEC cell line was purchased from the Pasteur Institute of Iran and was transferred in DMEM media enriched with 10% Foetal Bovine Serum (FBS, Gibco). The cells were then added into T25 cell culture flasks and incubated at a temperature of 37 °C in the presence of 5% CO2. After 3–5 days, confluency of the cells was assessed. At 90% of confluency, cells were removed from the flask by trypsin treatment until their use in following experiments.

### 2.6. Functional evaluation of the peptide and nanobody based on MTT method

About 10^4^ of HUVEC cells were suspended in 1 ml of culture media enriched with 2% FBS, and were then transferred to 96-well plates, followed by an incubation at a temperature of 37 °C for 2 h. After incubation at a temperature of 37 °C in the presence of 5% CO2, various concentrations of nanobody and peptide (0–1000 nM) were added to cells, followed by an incubation for 24 and 48 h, respectively. Bevacizumab was used as positive control. After incubation, an MTT (3–(4,5-Dimethylthiazol-2-yl)-2,5-Diphenyltetrazolium Bromide) solution was added to each well and incubated for 4 h. Thereafter, MTT was removed and DMSO (dimethylsulfoxide) was added to the wells. Plates were incubated on shaker for 30 min, and optical densities (OD) were then measured using a spectrophotometer (Epoch).

### 2.7. Functional evaluation of the peptide and nanobody based on the tube formation assay

About 50 µl of Geltrex LDEV lacking growth factor (Gibco, Invitrogen) were added to 96-well plate followed by a 30–60 min period of incubation at a temperature of 37 °C. One thousands nM of nanobody and peptide were added in two separate microtubes. Then, 50 ng/ml of VEGF were added to the tubes and incubated at a temperature of 37 °C for 1 h. Bevacizumab was as positive control. About 10^4^ of HUVEC cells with DMEM culture media plus each microtube’s mixture were seeded in 96-well plates containing Geltrex. Plates were incubated for 4–8 h (temperature of 37 °C in the presence of 5% CO2). After incubation, cells were assessed according to their conditions of tube formation, and tube-like structures analysed by Image J software.

### 2.8. Statistical analysis

Prism 5.0 Software (GraphPad, San Diego, CA) was used for statistical analysis. T test was performed for comparison between two groups. The statistical were considered significant when *p* values < .05.

## 3. Results

### 3.1. Bioinformatics and software analyses

**Figure 1.**
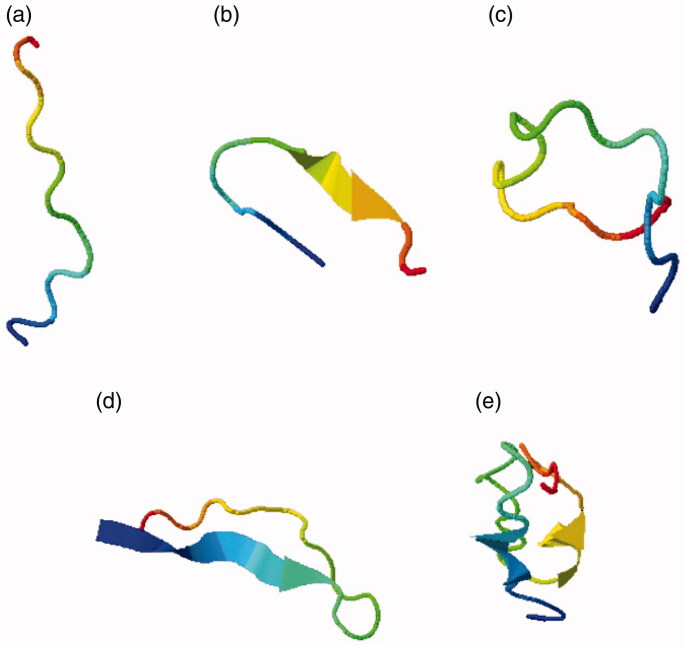
CDR structures of the VEGF nanobody obtained from I-TASSER. (a) CDR1 structure. (b) CDR2 structure. (c) CDR3 structure. (d) CDR1,3 structure. (e) CDR1,2 structure.

Different 3 D structure models were obtained by I-TASSER and their energies were minimised using Swiss modeller. In Table 1, different levels of energies of each predicted model were assessed. According to the structural models from I-TASSER, characteristics and number of amino acid residues of each structure were predicted. Figure 1 highlights the different CDR structures described by using I-TASSER. Table 2 shows the amino acid sequences that were submitted to I-TASSER whereas. Table 3 depicts the docking results of different nanobody structures. The data indicate that the binding energy of nanobody’s CDR3 region is similar to that of the entire nanobody. Further analysis strongly suggests that the CDR3 region would be a proper alternative regarding the interactions with VEGF.

**Table 1.** Results of computer-based energy minimizations on CDRs.

	Bonds	Angles	Torsion	Non bonded	Electro static	Constraint	Total
Nb	30/760	218/8	231/535	−423/64	−455/71	0	−548/62
CDR3	19/957	140/87	151/170	−333/46	−451/31	0	−438/24
CDR2	17/130	55/9	71/326	−206	−277/89	0	−277/87
CDR1	12/561	29/138	58/403	−128/15	−206/97	0	−183/54
CDR1,2	41/392	127/996	185/701	−341/80	−8/64	0	−34/93
CDR1,3	22/944	147/101	345/334	−635/45	−219/98	0	−300/12
CDR2.3	29/834	139/959	206/231	−570/65	−261/91	0	−425/22

‘Total’ indicates the most stable energy level of each structure obtained from Swiss modeller.

**Table 2.** Amino acid sequences of nanobody CDRs submitted to I-TASSER.

Peptides	Amino acid residues count	Amino acid sequences
CDR1	21	ASGFAYSTYSMG
CDR2	12	ATINSGTFRLWY
CDR3	19	AARAWSPYSSTVDAGDFRY
CDR1,2	21	GFAYSTYSGGGGGINSGTFRL
CDR2,3	32	INSGTFRLGGGGGAARAWSPYSSTVDAGDFRY
CDR1,3	30	GFAYTYSGGGGAARAMSPYSSTVDAGDFRY

**Table 3.** Results on docking simulation experiments of CDR regions and VEGF nanobody.

	E. Total (kcal/mol)	E. Shape	E. Air	Bmp (Bit maps)	Rms (Root mean square)
Nb	−342/15	−342/15	0	−1	−1
CDR3	−274/23	−274/23	0	−1	−1
CDR2	−167/2	−167/2	0	−1	−1
CDR1	−148/0	−148/0	0	−1	−1
CDR1,2	−160/5	−160/5	0	−1	−1
CDR1,3	−144/8	−144/8	0	−1	−1
CDR2,3	−162/1	−162/1	0	−1	−1

‘E. Total’ highlights the binding energy of a nanobody and its CDR3 region. The energy related to the CDR3 region is closest to that of the complete nanobody structure, as compared to other CDR regions.

### 3.2. Expression and purification of anti-VEGF nanobody

**Figure 2.**
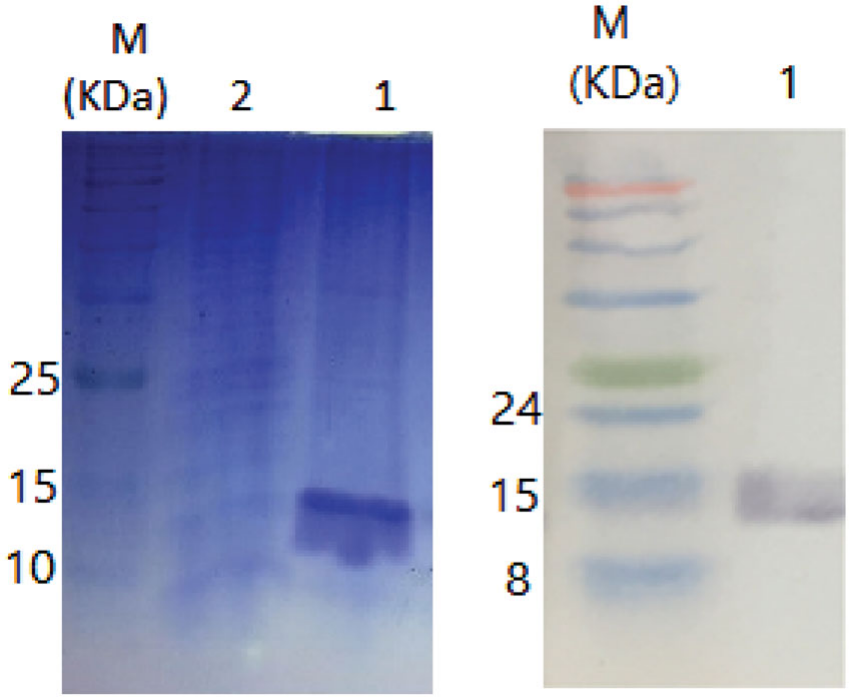
(a) SDS-PAGE of the purified nanobody. (b) Western blotting of the purified nanobody. M; protein marker, 1; the extracted nanobody 2; wash flow through.

The expression of nanobody was induced using various concentrations of IPTG (i.e., 0.3, 0.5, 0.7 and 1 mM). The highest level of expression was observed at 1 mM concentration (temperature of 37 °C for 12–14 h). Nickel affinity chromatography was performed for nanobody purification. The purification step was checked by SDS-PAGE and Western blotting. Figure 2 illustrates the purification of nanobody (15 kDa bands are indicative of the purified nanobody).

### 3.3. Affinity results

Affinity of peptide, nanobody, and Bevacizumab to VEGF was calculated by ELISA method of Beatty et al.^34^. Affinity constant of peptide and nanobody were 51 × 109 M^−1^ and 60 × 109 M^−1^, respectively. In addition, calculated affinity for Bevacizumab was 56 × 1012 M^−1^.

### 3.4. Functional evaluation of peptide and nanobody based on the MTT method

**Figure 3.**
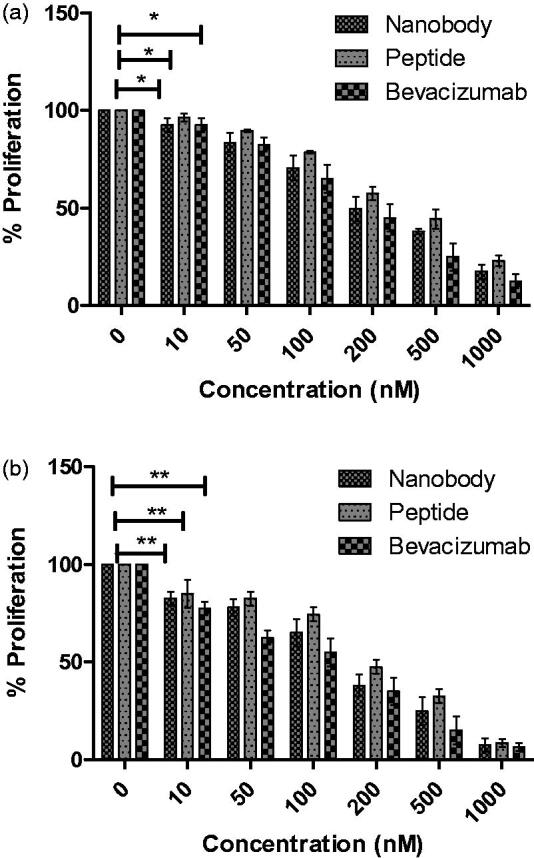
MTT assay results. (a) The effects of nanobody and peptide on the growth of HUVEC cells after 24 h and (b) 48 h. Determined IC_50_s(24 h) were 200, 300, and 350 nM for bevacizamab, nanobody, and peptide, respectively. Moreover, calculated IC_50_s(48 h) were 150, 170, and 200 nM for bevacizamab, nanobody, and peptide, respectively. Data are presented as mean ± SD. *p value s= .0292, **p values = .001.

MTT is a yellow soluble substance which is reduced to purple formazan by mitochondrial dehydrogenase enzyme in living cells. DMSO degrades cellular membrane and reportedly dissolves formazan^35^. Different methods are available to evaluate the effects of angiogenic inhibitors, including cellular proliferation and migration assays. In this study, two assays were used to investigate the function of nanobody and peptide. The MTT assay was used to evaluate the inhibitory effects of nanobodies and peptide on the proliferation of endothelial cells^36^. As shown in Figure 3, increasing the concentration of nanobody and peptide resulted in greater inhibition of cellular proliferation. Therefore, effects of both compounds are dose dependent. In addition, by investigating the time dependency of drug’s effects, we showed that the nanobody and peptide are also time dependent. Analyses of 24- and 48-h treatment of nanobody and peptide on HUVECs, indicate a *p* values less than 0.05. According to MTT data, inhibition of cell growth was observed in almost all cells in concentration of 1000 nM. At such compound’s concentration (1000 nM), inhibitions of cell growth were respectively of 83 and 92% after 24- and 48-h incubation in the assay with nanobody, whereas they were respectively of 77 and 91% after 24- and 48-h incubation in the assay with peptide. However, inhibitions of cell growth were 87 and 97% after 24- and 48- h incubation with Bevacizumab, respectively. Determined IC_50_ s(24 h) were 200, 300, and 350 nM for bevacizamab, nanobody, and peptide, respectively. Moreover, calculated IC_50_s(48 h) were 150, 170, and 200 nM for bevacizamab, nanobody, and peptide, respectively.

**Figure 4.**
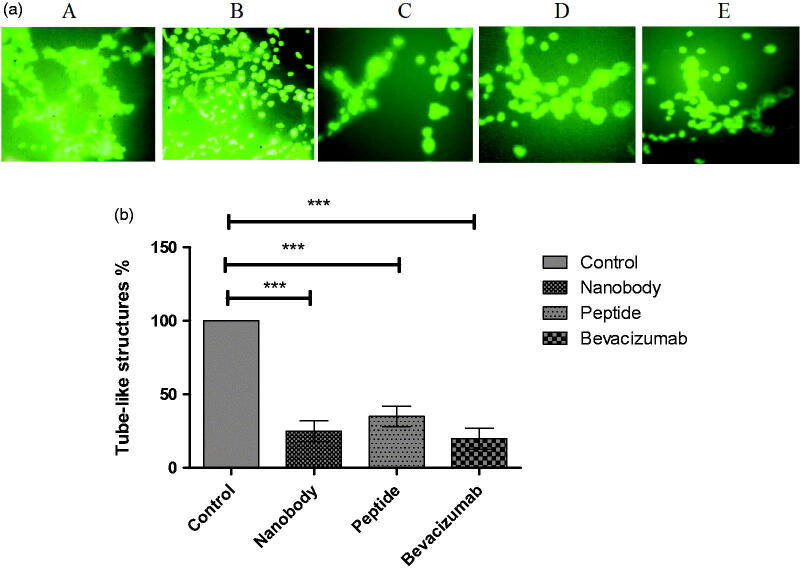
Tube assay results. (a) Tube like structures: A; HUVEC cells with VEGF, B; without VEGF, C; Nanobody, D; Peptide, E; Bevacizumab. (b) Quantification of tube assay results. Control; HUVEC cells in the presence of VEGF and without any inhibitory factors formed complete tube-like structures. HUVEC cells in the presence of nanobody, peptide, and Bevacizumab formed 25, 35, and 20% of tube-like structures, respectively. Data are presented as mean ± SD. ***; *p* values = .0001.

### 3.5. Functional evaluation of peptide and nanobody with tube formation assay

Many studies have shown that the presence of VEGF and its binding to the cell surface receptors activate a signalling cascade which leads to cell proliferation, differentiation and tube formation^23^. This assay was therefore conducted to point out the ability of nanobody and peptide to inhibit VEGF, and consequently inhibit tube formation. As evidenced experimentally, only tube-like structures were completely formed in wells containing HUVEC cells treated with VEGF (control well) (Figure 4a). However, percentages of tube-like structures were 25, 35, and 20 in cells treated with nanobody, peptide, and Bevacizumab, respectively (Figure 4b).

## 4. Discussion

Angiogenesis is a vital process through which tumour cells grow. A current approach of tumour treatment relies on angiogenesis inhibition. One of the ‘key’ factors in forming new blood vessels is VEGF. The inhibition of VEGF, and the associated blockade of its signalling pathway (pathway which depends on VEGF binding to its cell receptors), are effective steps towards cancer treatment^37^^,^^38^. Antibodies are used as anti-angiogenesis drugs. By developing the field of antibody engineering, one may reasonably suggests that a new generation of therapeutic molecules will emerge^39^^,^^40^. This new generation of molecules may well be represented by nanobodies. They can be characterised by their high affinities towards the targets, molecular weights of 15 kDa, and lower production costs as compared to those of ‘classic’ antibodies^13–15^^,^^27^^,^^41^^,^^42^. Nanobodies can infiltrate in tumour tissues due to their small size and single domain structure. Nanobodies have regions/domains called CDRs that enable them to recognise distinct epitopes, small pits and grooves which are not recognisable by ‘regular’ antibodies^41^^,^^43^. Therefore, it appears that designing and producing ‘new’ peptides mimicking parts of larger drug molecules would be essential to get candidate chemotherapeutic compounds with appropriate structural features and functional properties^44^. Because of the established ‘key’ role of VEGF in angiogenesis and according to the appropriate/favorable nanobody’s characteristics^5^^,^^28^^,^^45^, we aimed at designing a mimotope that can play nanobody’s role in the present study. Here, for the first time we investigated whether the CDR3 domain of the VEGF nanobody mimicked by a peptide can act as a ligand of VEGF and inhibit interactions of VEGF with its cell receptors. By using several dedicated softwares, we examined the CDR structures of nanobody and found that the CDR3 region of nanobody might behave as an entire VEGF nanobody. According to our analyses on the whole nanobody, its CDR regions and VEGF receptor, we found that the CDR3 region with 20 amino acid residues had an affinity greater than that of the nanobody itself. Therefore, we used CDR3 in our study instead of a complete nanobody. Nanobody’s CDR3 can directly bind to an antigen. Binding of CDR3 to specific amino acid residues results in the proper configuration of nanobody. A disulphide bond between the CDR1 and CDR3 regions is expected to maintain the stability of molecule in particular situations, such as high temperature, presence of protease(s), and acidic environment^20^. A study focussing on various CDR3 regions highlighted that some constant hydrophobic amino acid residues do exist in all CDR3s. According to previous studies, CDR3-related amino acid residues reportedly play an important role in the structural/functional maintenance of the VHH domain. Experiments on the CDR3 domains have shown that there are some repetitive motifs in all CDR3s, which create a positive load in the CDR3 structures^21^. In a next step, we expressed and extracted a previously developed VEGF nanobody^5^ to investigate the difference between nanobody’s and peptide’s effects. The final expression level of VEGF nanobody in *E. coli* wk6 cells was 5 mg/L. To investigate the functional effects of the nanobody and peptide, we used MTT and tube formation assays. Our data demonstrate that both nanobody and peptide were able to inhibit cell proliferation. The effects of peptide on inhibition of cell proliferation were near to the effects observed with the nanobody.

Tube formation assay was performed to evaluate the effects of peptide and nanobody. The tube formation assay is the first, most important and relevant one to investigate the effects of a compound on angiogenesis. This test is actually performed in almost all of the reported studies investigating candidate angiogenic compounds^23^^,^^46^. The tube formation assay is a fast and quantitative. The tube formation assay can ‘dissect’ the various angiogenesis stages, such as cell adherence, cell migration, cell alignment, and tube formation. This test is reportedly sensitive, reliable and fast^23^. The results showed that increasing the concentration of peptide or nanobody, linearly decreases the proliferation of cells. Interestingly, the effects of peptide appear to be almost similar to that of the nanobody. As expected, the designed peptide was also able to inhibit tube formation in HUVEC cells.

Different mimotopes have been designed by various approaches for cancer treatment. For example, a study focussed on a mimotope mimicking specific epitopes that induced antibodies against VEGF. Of note, this mimotope was established using the phage display method^47^. In another study, mimotopes (with low molecular weights) were expressed on the surface of phage particles and were used as a substitute of natural EGFR to induce an ‘active’ immunity responsible for a long term humoral response^48^. In an additional study, a mimotope was extracted from tocilizumab which could induce dual humoral and cellular responses of the immune system^49^. Pourhashem et al. also designed a mimotope against HER3 using *in silico* studies, but not fully characterised and requiring further *in vitro* and *in vivo* experiments^24^.

Due to their advantages, mimotopes are used in cancer immunotherapy studies instead of natural epitopes. Mimotopes have short linear amino acid sequences and are ‘easy’ to produce, which makes them suitable substitutes of natural epitopes. Mimotopes can even be synthesised for undetected epitopes, because knowledge of the antigenic amino acid sequence is not required for their production^48^. Different research teams have produced a variety of mimotopes for different disorders and have used distinct approaches. To the best of our knowledge, the current study is the first one relying on bioinformatics to produce a VEGF nanobody-mimicking peptide, and can be therefore considered as a starting point of future studies in the field.

## Conclusion

In this study, we designed a peptide derived from the CDR3 region of a VEGF nanobody that targets VEGF. According to our data, the CDR3 region of nanobody has an affinity very similar to that of a complete nanobody against VEGF. The synthesised peptide significantly inhibited cell proliferation and angiogenesis *in vitro*. These results highlight the potential of a mimotope designed from the structure of its nanobody to inhibit a pathophysiological process such as cancer angiogenesis.
